# The impact of insulin resistance on thyroid function and the prevalence of thyroid follicular nodular disease in pregnant women

**DOI:** 10.1530/ETJ-24-0317

**Published:** 2025-04-03

**Authors:** Andrzej Nowak, Jacek Podlewski, Alicja Hubalewska-Dydejczyk, Małgorzata Trofimiuk-Müldner

**Affiliations:** ^1^ Medical University of Warsaw, Department of Gynecological Endocrinology, Warszawa, Poland; ^2^ Dover Fueling Solutions, Kraków, Poland; ^3^ Jagiellonian University Medical College, Chair and Department of Endocrinology, Kraków, Poland

**Keywords:** insulin resistance, thyroid, pregnancy, HOMA-IR, nodularity

## Abstract

**Introduction:**

Insulin resistance (IR) is a phenomenon commonly observed in pregnancy. Increased insulin concentrations might impact thyroid function and structure during gestation.

**Objectives:**

This study investigates the bidirectional relationship between IR indices and thyroid function and morphology in pregnant women.

**Methods:**

In 1,069 gravid participants of the Polish National Programme for Elimination of Iodine Deficiency (2007–2017), blood samples were analyzed for thyroid-stimulating hormone (TSH), FT3, FT4, aTPO, fasting glucose and insulin concentrations, and the thyroid structure was assessed with ultrasound (in 1,065 subjects). Based on calculated homeostatic model assessment of insulin resistance (HOMA-IR) values, participants were stratified into two subgroups: HOMA-nl (HOMA-IR <2.5) and HOMA-h (HOMA-IR ≥2.5), comprising 894 and 175 women, respectively.

**Results:**

Significant difference in mean TSH (1.77 ± 1.17 vs 1.96 ± 1.04; *P* = 0.008) and mean FT4 (12.65 ± 2.3 vs 11.47 ± 1.9; *P* = 0.001) concentrations between HOMA-nl and HOMA-h groups was found. The subgroups did not differ in thyroid nodularity or multinodular goiter prevalence. HOMA-IR positively correlated with TSH concentrations, BMI and thyroid volume. Serum FT3 and FT4 concentrations showed negative correlations with HOMA-IR.

**Conclusions:**

IR seems to affect the thyroid function of gravid women by diminishing the ability to respond to increased thyroid hormone demand. Thyroid volume increase during pregnancy may be influenced by IR; however, its short-term effect on thyroid nodularity appears to be negligible.

## Introduction

Insulin resistance (IR) is defined as a decreased sensitivity of muscles, adipose tissue, liver and other body tissues to insulin despite its normal or increased concentration in blood (1, 2). IR may predispose to or accompany a variety of conditions clustered within metabolic syndrome.

IR is also a physiological phenomenon during pregnancy (3), primarily due to the actions of pregnancy-related hormones such as human placental lactogen, progesterone and cortisol, which can interfere with insulin signaling pathways. IR increases gradually throughout pregnancy, peaking in the late second or early third trimester. This is believed to be an adaptive response to ensure an adequate supply of nutrients to a growing fetus. However, excessive IR during pregnancy can lead to gestational diabetes mellitus (GDM), a condition that develops or is recognized during pregnancy (3, 4).

Many factors modify maternal thyroid function during pregnancy, including placental hormones, iodine nutrition, ethnicity and body mass index (BMI) (5, 6). It remains to be answered whether disturbances in carbohydrate metabolism, including IR, can affect the function or structure of the thyroid gland and vice versa. Insulin, as an anabolic hormone, may be responsible for thyroid hypertrophy and nodular development (2, 5). There is evidence suggesting that thyroid disorders increase the risk of type 2 diabetes (T2D) mellitus (6).

This study aimed to evaluate the association between TH and structure, as well as IR in pregnant women.

## Materials and methods

### Study group

The study was conducted between 2007 and 2017 as part of the Polish National Programme for Elimination of Iodine Deficiency in randomly selected towns and cities in Poland with at least one maternity ward. The patients were recruited *via* maternity wards, obstetricians’ practices and social media.

**Figure 1:**
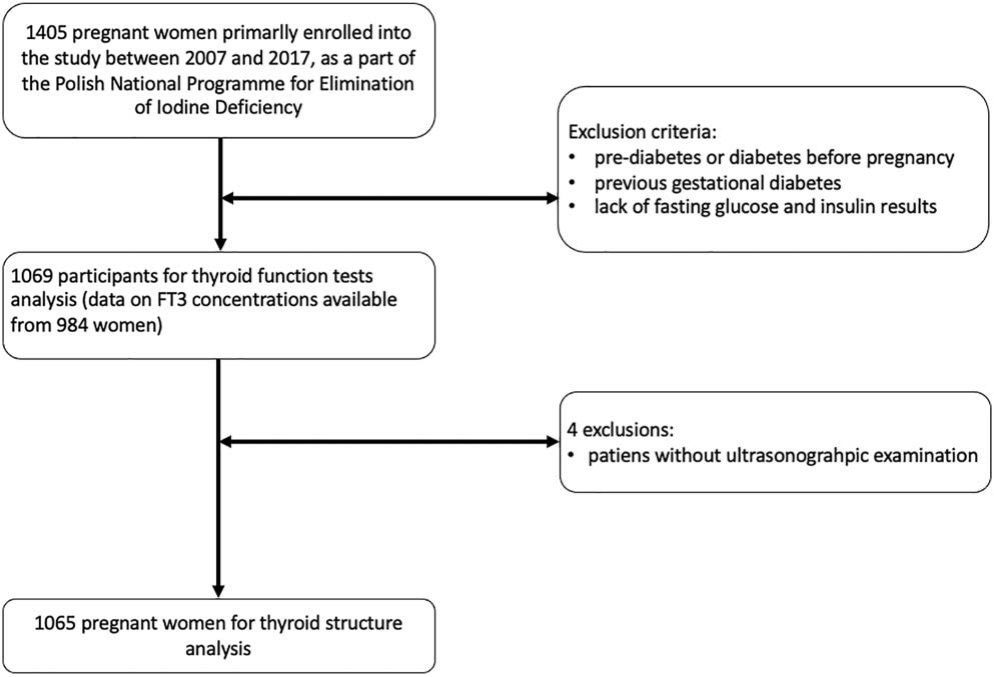
Flowchart of participants' selection for thyroid function and structure analysis in pregnant women.

The primary study group comprised 1,405 apparently healthy pregnant volunteers – 213 (16%), 531 (40%) and 595 (44%) in the 1st, 2nd and 3rd trimester of pregnancy, respectively. After the exclusion of participants without fasting glucose and insulin measurements, patients with pre-diabetes, diabetes before pregnancy and patients with previous gestational diabetes, 1,069 subjects (median age (interquartile range IQR): 29 (6) years) were included for thyroid function analysis (173 (16.2%) in the 1st trimester, 488 (45.6%) in the 2nd trimester and 408 (38.2%) in the 3rd trimester of pregnancy) and 1,065 for thyroid structure analysis (Fig. 1). Written informed consent was obtained from each participant. The study was approved by the local Ethics Board (1072.61201.7.2017).

### Methods

Each participant was asked to fill in a questionnaire regarding maternal age, gestational age, the number of previous pregnancies and miscarriages, comorbidities, medications and supplements use. During recruitment, BMI was calculated based on the participant’s height and weight according to the formula in metric units: weight (kilograms) divided by height squared (meters) (7).

Blood samples for thyroid-stimulating hormone (TSH), FT4, aTPO, fasting glucose and insulin were obtained from each included volunteer. Blood samples for FT3 concentration assessment were collected from 984 participants (68.6% of the study group). Whole blood samples were collected in standard no-additive tubes and centrifuged at room temperature. Serum aliquots were pipetted to storage vials, frozen and stored at −20°C until measurements (usually within 7 days from blood retrieval). Throughout the study period, blood samples were analyzed in the Clinical Biochemistry Department of the University Hospital in Krakow with the ROCHE Cobas® 6000/8000 platform by electrochemiluminescence (ECL) (Roche Elecsys TSH assay, Roche Elecsys FT4 II assay, Roche Elecsys FT3 III assay, Roche Elecsys anti-TPO assay and Roche Elecsys Insulin assay (Roche Diagnostics, Germany) for TSH, FT4, FT3, ATPO and insulin respectively). Serum glucose was measured by the hexokinase method. The Clinical Biochemistry Department is certified by the Randox International Quality Assessment Scheme, the Unity Interlaboratory Comparison Program, the Sysmex International Quality Assurance System and the StandLab IQS. Quantification limits for TSH were 0.005–100 mIU/L, FT3: 0–50 pmol/L, FT4: 0–100 pmol/L and anti-TPO: 5–600 IU/mL. The reference ranges for TH assays for non-pregnant adults were 0.3–4.5 mIU/L for TSH, 11–22 pmol/L for FT4, 3.1–6.8 pmol/L for FT3, 3.30–5.60 mmol/L for glucose and 2.6–24.9 μU/mL for insulin. ATPO positivity was defined according to the manufacturer as ATPO concentrations ≥34 IU/mL.

HOMA-IR (homeostatic model assessment of insulin resistance) was calculated as the fasting insulin concentration (in μU/mL) multiplied by the fasting glucose concentration (in mmol/L) and divided by 22.5 (8). In each study participant, urinary iodine concentration (UIC) measurements were performed in casual morning urine samples by the Sandell-Kolthoff method in the Clinical Biochemistry Department of the Jagiellonian University Medical College and in the Medical Diagnostics Unit of the Faculty of Pharmacy of the Jagiellonian University Medical College in Krakow according to the WHO and the EUthyroid Consortium recommendations (9).

A thyroid ultrasound was performed once in 1065 study participants. The examinations were conducted according to the recommendations of the Polish Society of Ultrasonography (10) using the Siemens Sonoline ultrasound system equipped with a 7.5 MHz linear probe, which was provided by Merck KGaA, Darmstadt, Germany. The thyroid volume (TV) was calculated using Brunn’s formula, which involves measurements of the right lobe (RL) and left lobe (LL) widths, depths and lengths in centimeters (TV (mL) = 0.479*(RL width (cm)*RL depth (cm)*RL length (cm)) + 0.479*(LL width (cm)*LL depth (cm)*LL length (cm)‏) (11).

According to the norms for the female Polish population, a TV greater than 20 mL was considered to be a goiter (12). The echogenicity of the thyroid parenchyma was compared to the echogenicity of surrounding tissues. The presence, number and size of thyroid nodules were recorded.

### Statistical analysis

Statistical analysis was performed using R package version 4.2.2 (R Core Team, 2022). The ggplot2 package (Wickham 2016) was used to create graphs.

All the TH and UICs showed right-skewed distributions. For FT4 and TSH, normality was achieved by logarithmic transformation. The HOMA subgroups were compared using one-way ANOVA and Tukey’s HSD test. For other parameters, Kruskal–Wallis and Dunn’s post hoc tests were used to compare subgroup differences, as these are rank-based methods that do not require the normality of distributions. HOMA values between ATPO-positive and ATPO-negative patients were compared using the Mann–Whitney U test. Because of a normal distribution with heterogeneous variance (as shown by Levene’s test) of TVs in HOMA subgroups, Welch’s ANOVA and Games-Howell post hoc tests were applied for analysis of between-subgroup differences, using the rstatix R package. Receiver operating characteristics (ROC) curve analysis and cut-off point search were performed with the cutpointr R package (12, 13). AUC (area under curve) serves here as a measure of the overall predictive power of HOMA in explaining the presence of thyroid nodules without selecting a specific cut-off. AUC values close to 1 indicate that it is possible to accurately predict thyroid nodularity using HOMA, while a value of 0.5 means that a prediction is no better than random. To find possible cut-off points, we used an algorithm that screens the range of HOMA values and finds one such that the resulting ‘above’ and ‘below’ subgroups maximize some measure of association with thyroid nodules. Out of many available methods, we applied the chi-square test, F1 score and the Youden index to calculate the *P*-value.

For all the frequency tables, differences between subgroups were assessed using Pearson’s chi-square test or Fisher’s exact test in the case of subgroups with very low counts (any subgroup with less than five subjects). The significance of correlations was checked using a standard *t*-test with Fisher’s Z-transformation. Multivariate regression models were built to assess relationships controlled for other factors. Reported results came from ordinary least squares (OLS) regression, but bootstrap analysis was also conducted to confirm the validity of the conclusions drawn.

## Results

Based on calculated HOMA-IR, participants were stratified into two subgroups: HOMA-nl (HOMA-IR values <2.5) comprised 894 women (83.6% of the study group) and HOMA-h (HOMA-IR values ≥2.5) comprised 175 women (16.4% of the study group). The subgroups’ characteristics are shown in Table 1.

**Table 1:** Characteristics of the HOMA-nl and HOMA-h subgroups. Data are presented as mean ± SD or as *n* (%).

	HOMA-nl	HOMA-h	*P* value	Total
Age (years)	29.04 ± 4.87	28.9 ± 4.92	0.79	29.03 ± 4.87
BMI (kg/m^2^)	22.53 ± 3.4	25.37 ± 4.73	<0.001	23 ± 3.8
Pregnancy week	21.6 ± 8.48	26.93 ± 8.66	<0.001	22.48 ± 8.74
Iodine supplementation			0.109	
No	345 (38.7%)	56 (32%)		401 (37.7%)
Yes	545 (61.3%)	119 (68%)		664 (62.3%)
Number of pregnancies			0.271	
1	465 (52.2%)	80 (45.7%)		545 (51.2%)
2	252 (28.3%)	58 (33.1%)		310 (29.1%)
3+	173 (19.4%)	37 (21.1%)		210 (19.7%)
Complications during previous pregnancies			0.278	
NA	465 (52.2%)	80 (45.7%)		545 (51.2%)
No	254 (28.5%)	58 (33.1%)		312 (29.3%)
Yes	173 (19.2%)	37 (21.1%)		208 (19.5%)

BMI, body mass index; SD, standard deviation.

The TH, ATPO and UICs for each subgroup are presented in Table 2.

**Table 2:** Thyroid hormones, ATPO and UICs in study subgroups.

	HOMA-nl	HOMA-h	*P* value
ATPO (IU/mL)			0.037
Mean (SD)	30.47 (75.07)	22.66 (49.96)	
Median (IQR)	10.95 (9.47)	10.15 (7.5)	
FT3 (pmol/L)			0.032
Mean (SD)	4.89 (1.07)	4.66 (0.9)	
Median (IQR)	4.66 (1.18)	4.47 (0.88)	
FT4 (pmol/L)			<0.001
Mean (SD)	12.65 (2.3)	11.47 (1.9)	
Median (IQR)	12.48 (2.85)	11.38 (2.8)	
TSH (mIU/L)			0.008
Mean (SD)	1.77 (1.17)	1.96 (1.04)	
Median (IQR)	1.57 (1.32)	1.77 (1.29)	
UIC (μg/L)			0.099
Mean (SD)	114.67 (78.19)	101.26 (64.5)	
Median (IQR)	93.28 (81.87)	87.1 (63.23)	

ATPO, antibodies against thyroid peroxidase; IQR, interquartile range; FT3, free triiodothyronine; FT3, free thyroxine; SD, standard deviation; TSH, thyroid-stimulating hormone; UIC, urinary iodine concentration.

There was a significant difference in mean ± SD TSH: 1.77±1.17 vs 1.96±1.04; *P* = 0.008, mean FT4: 2.65±2.3 vs 11.47±1.9; *P* = 0.0001 and mean FT3: 4.89±1.07 vs 4.66±0.9; *P* = 0.032 concentrations between HOMA-nl and HOMA-h subgroups. Furthermore, the subgroups differed significantly in mean±SD ATPO concentrations: 30.47±75.07 vs 22.66±49.96; *P* = 0.037, but there was no significant difference in the prevalence of ATPO-positivity in the HOMA-nl and HOMA-h subgroups (125 patients (14%) vs 16 patients (9%); *P* = 0.1037). There was also no significant difference in mean±SD HOMA-IR values between volunteers with ATPO <34 and ≥34: 1.80±2.01 and 1.63± 1.50, respectively, *P* = 0.32. The subgroups did not differ in UIC concentrations (Table 2).

### Thyroid structure

Mean±SD TV was 12.58±5.00 mL and 13.7±4.92 mL for HOMA-nl and HOMA-h subgroups, respectively (*P* = 0.0012).

At least one thyroid nodule was found in 212 (20%) of the investigated subjects. There was no significant difference in thyroid nodularity frequency between the subgroups (20.6% (183 subjects) and 16.6% (29 subjects) in HOMA-nl and HOMA-h, respectively; *P* = 0.27), as well as in the proportion of multinodular goiter between the subgroups (*P* = 0.61). There was also no significant difference in thyroid nodularity between the subjects with HOMA-IR values ≥4 and <4 (*P* = 0.74) (Table 3).

**Table 3:** Thyroid nodularity according to HOMA-IR cut-offs. Data are presented as *n* (%).

Nodules, *n*	HOMA-IR cut-off			
	HOMA <2.5	HOMA ≥2.5*	HOMA <4	HOMA ≥4**
0	707 (79.4)	146 (83.4)	806 (80)	47 (82.5)
≥1	183 (20.5)	29 (16.6)	202 (20)	10 (17.5)

* HOMA<2.5 vs HOMA ≥2.5: *P* = 0.27.

** HOMA <4 vs HOMA ≥4: *P* = 0.74.

HOMA-IR, homeostatic model assessment of insulin resistance.

As neither HOMA-IR ≥2.5 nor HOMA-IR ≥4 was related to the prevalence of thyroid nodular disease, we searched the range of observed HOMA-IR values for optimal cut-off values for predicting the presence of thyroid nodules. However, ROC analysis failed to provide such HOMA-IR threshold, as it suggested no predictive relationship between HOMA-IR and thyroid nodules (AUC = 0.482, *P* = 0.79).

No significant differences between the subgroups in the prevalence of abnormal echogenicity were found (18% and 17.1% for HOMA-nl and HOMA-h, respectively; *P* = 0.83).

### Correlations between HOMA-IR and pregnancy-related thyroid function and thyroid structure parameters

In our study group, HOMA-IR correlated positively with serum TSH concentrations, BMI and TV (Table 4). Serum FT3 and FT4 concentrations negatively and strongly correlated with HOMA-IR (Table 4). Neither UIC nor ATPO concentrations correlated significantly with HOMA-IR.

**Table 4:** Correlations between HOMA-IR and analyzed parameters.

Parameter	*r*	*P* value	*n*
BMI	0.224	<0.001	1,055
TSH	0.072	0.02	1,065
FT3	−0.102	0.01	931
FT4	−0.138	<0.001	1,065
UIC	−0.057	0.07	1,012
TV	0.062	0.04	1,065
ATPO	−0.015	0.61	1,065
Pregnancy week	0.244	<0.001	1,065
Number of thyroid nodules	−0.025	0.41	1,065
FT3/FT4 ratio	0.023	0.49	931

ATPO, antibodies against thyroid peroxidase; BMI, body mass index; IQR, interquartile range; FT3, free triiodothyronine; FT3, free thyroxine; HOMA-IR, homeostatic model assessment of insulin resistance; *n*, number of participants; *r*, correlation coefficient; SD, standard deviation; TSH, thyroid-stimulating hormone; UIC, urinary iodine concentration; TV, thyroid volume.

We assessed the isolated effect of IR on TSH, FT4 and TV while controlling for BMI and pregnancy week with multivariate regression analysis. HOMA-IR discretized into two categories (<2.5, ≥2.5), maintained a significant relationship with FT4 (*P* = 0.027), while it was no longer a significant predictor for TSH (*P* = 0.14) and TV (*P* = 0.91) after controlling (Table 5).

**Table 5:** HOMA-IR relationship with TSH and FT4 concentrations and TV adjusted to confounders (BMI, pregnancy week).

	Coefficient	SE	*t* (1,051)	*P* value
Adj HOMA on TSH				
Intercept	1.6877	0.2349	7.1857	<0.001
HOMA≥2.5	0.1482	0.1002	1.4789	0.139
BMI	−0.0095	0.0096	−0.9892	0.322
Week	0.0136	0.0041	3.3124	0.001
Adj HOMA on FT4				
Intercept	17.0185	0.408	41.7167	<0.001
HOMA ≥2.5	−0.3856	0.174	−2.2153	0.027
BMI	−0.0671	0.0166	−4.0385	<0.001
Week	−0.128	0.0071	−17.9281	<0.001
Adj HOMA on TV				
Intercept	4.9521	1.0049	4.9277	<0.001
HOMA ≥2.5	−0.0466	0.4287	−0.1087	0.913
BMI	0.2537	0.0409	6.1992	<0.001
Week	0.0888	0.0176	5.0475	<0.001

Adj, adjusted effect of; BMI, body mass index; HOMA-IR, homeostatic model assessment of insulin resistance; SE, standard error; t, *t*-test statistic; TV, thyroid volume.

Our dataset has some characteristics that could potentially have skewed the OLS regression results – BMI, TV and TSH distributions having heavy tails and discretized HOMA being imbalanced (only 16.4% of patients in the ≥2.5 class). To check for potential issues, we calculated variance inflation factors (VIFs) for predictors and re-estimated all models using bootstrap analysis, which is known to better adapt to arbitrary data distributions. All VIFs were close to 1, while values above ten typically indicate a problem. Bootstrap regression models produced very similar results to OLS, with the same variables being significant. Hence, we only report the OLS regression results, being confident about the analysis’s validity.

## Discussion

TH plays a role in glucose utilization in muscles, hepatic glucose output and splanchnic glucose absorption, thus impacting insulin sensitivity (and, potentially, a risk of impaired carbohydrate metabolism) (14).

Thyroid disorders have been found to potentially increase susceptibility to T2D, as indicated by a comprehensive study conducted on a national scale (6, 15). With a follow-up period of 10 years, Chen *et al.* demonstrated a significantly greater incidence of T2D in the thyroid dysfunction group compared to the control group, with an adjusted HR of 1.23 (95% CI: 1.16–1.31). The adjusted HR of developing T2D for hypothyroid subjects was 1.19 (95% CI: 1.07–1.33). Females (HR: 1.27, 95% CI: 1.18–1.37), persons aged 18–39 years (HR: 1.51, 95% CI: 1.32–1.73), and subjects without comorbidities (HR: 1.47, 95% CI: 1.34–1.60) were more prone to be diagnosed (6). According to Dueñas *et al.* meta-analysis, the pooled HR of T2D was 1.26 (95% CI: 1.05–1.52) for hypothyroid patients (15).

Lower FT4 within the reference range has been linked to an increased risk of T2D (15). The lowest FT4 concentrations were related to the higher prevalence of metabolic syndrome in a large South Korean study on healthy euthyroid subjects; however, such a trend disappeared after adjustment for age regardless of the gender (16). Such observations are not consistent. For example, FT4 and FT3 levels were positively correlated with T2D risk in the Study of Health in Pomerania (SHIP) cohort (17). Kus *et al.* (18) have found no evidence of an association between FT4 levels and T2D risk in a Mendelian randomization study. Primary analyses also failed to reveal an association between TSH and T2D risk; however, after excluding pleiotropic instruments, the authors noted a significant association between higher TSH concentrations and T2D risk.

Overt hypothyroidism has been linked to changes in glucose metabolism and insulin sensitivity (14, 15). A drop in adrenergic activity observed in hypothyroid subjects may result in decreased glycogenolysis, gluconeogenesis and basal insulin secretion. Increased leptin secretion, changes in GLUT4 translocation and elevated serum free fatty acids were postulated to contribute to IR in hypothyroidism (19). Kim *et al.* (20) described the synergistic role of T3 and insulin in maintaining glucose homeostasis, at both the cellular and molecular levels. They hypothesized that the impaired glucose disposal seen in IR may be attributed to a decrease in intracellular concentrations of serum T3. Lekakis *et al.* (21) suggested that impaired flow-mediated endothelial vasodilation observed in hypothyroid patients may contribute to the development of IR. A series of small clinical studies showed higher insulin concentrations in hypothyroid patients (22, 23, 24, 25). Subclinical hypothyroidism has also been linked to higher insulin concentrations and HOMA-IR values (24, 26). In a Dutch study (PREVEND cohort), low-normal FT4 levels were significantly associated with increased IR (27). However, mechanisms other than IR behind increased metabolic syndrome prevalence in a euthyroid population with low-normal FT4 concentrations have been suggested (16).

It has been proposed that thyroid dysfunction may play a role in the etiology of GDM. Pregnant patients diagnosed with GDM were found to have higher TSH and FT3 concentrations, lower FT4 concentrations and a higher FT3:FT4 ratio (28). Higher FT3 to FT4 ratios were found to be GDM risk factors in a study by Rawal *et al.*, the authors failed to find such a link for TSH and FT4 concentrations (29). TSH levels in the first trimester, even when they were within the normal range, were positively correlated with the risk of GDM in pregnant Chinese women, particularly in those who were overweight or obese before becoming pregnant (30). A recent Danish study (Odense Child Cohort) revealed that third-trimester HbA1c, fasting glucose, insulin and HOMA-IR are higher among women with lower early pregnancy FT4 concentrations (below the 25th percentile for the cohort). The inverse association between HbA1c and FT4 was the closest for the high-normal concentrations of FT4 (31).

In our study, lower TSH and higher FT4 and FT3 concentrations were observed in pregnant women with lower HOMA-IR values (<2.5), and a significant positive correlation between HOMA-IR and TSH concentrations and BMI was also confirmed.

It has been suggested that pregnant women with thyroid autoimmunity (TAI) have an increased risk of GDM (28, 32). In a study by Yanachkova & Kamenov (28), a significantly higher frequency of GDM was noted in ATPO-positive euthyroid women and women with isolated hypothyroxinemia (no such difference was observed in hypothyroid women, both subclinical and overt). A recent study by Sitoris *et al.* (32) explored the relationship between thyroid function tests (including ATPO) at a median 13 weeks of gestation and the risk of GDM (diagnosed based on an oral glucose tolerance test (OGTT) performed between 24 and 28 weeks of pregnancy). There was no significant difference in either ATPO concentrations or the frequency of TAI between pregnant women with and without GDM. A significant association (adjusted odds ratio: 1.68 (95% CI: 1.01–2.78; *P* = 0.048) was found between TAI and GDM risk only in the logistic regression model, in which age and BMI were expressed as categorical variables (>30 years and ≥30 kg/m^2^, respectively). In our study, a significant difference was observed in mean ATPO concentrations between the HOMA-nl and HOMA-h subgroups (30.47 ± 75.07 vs 22.66 ± 49.96; *P* = 0.037), the mean ATPO concentration in both groups was within the normal range. Similarly, we did not find a significant difference in the prevalence of ATPO positivity between these subgroups (*P* = 0.1037). Furthermore, we did not observe a statistically significant correlation between HOMA-IR values and ATPO concentrations (*P* = 0.61). Therefore, we suggest that the relations observed in our study are generally independent of TAI. It needs to be stressed that differences in methodology (we did not have access to OGTT results of those subjects who were examined in the first or early second trimester of pregnancy) might affect the analysis outcomes.

IR or increased HOMA-IR levels during pregnancy may not necessarily reflect reduced insulin sensitivity before conception. During the latter part of pregnancy, the mother’s ability to respond to insulin diminishes, which is considered a natural phenomenon. In our study, we managed to prove a significant negative correlation between HOMA-IR and FT4 concentrations, which was independent of gestational age and other factors. This observation may suggest an independent link between IR and hypothyroidism in pregnant women. Similar findings were reported in a recent study (not including pregnant women) in which lower FT4 levels were associated with increased IR (27).

Insulin, being an anabolic hormone, has been shown to have a mitogenic effect on thyroid cell cultures (33). Therefore, IR-related compensatory hyperinsulinemia appears to play a role in the development of thyroid nodules and goiter (2, 34). Obesity and metabolic syndrome, which are related to IR, have been linked to the increased prevalence of nodular thyroid disease (34, 35, 36, 37, 38, 39). IR itself may be a risk factor of goiter or/and thyroid nodular disease. In the study by Rezzonico *et al.* (40), both lean and obese subjects with IR have higher TV and prevalence of thyroid nodularity compared to their counterparts without IR.

In our group of pregnant women, we found a significant positive correlation between TV and HOMA-IR. This is consistent with the results of other research groups. Yasar *et al.* (5) and Lomtadze *et al.* (41) also documented a positive correlation between HOMA-IR and TV, although in a non-pregnant population.

We failed to prove a link between HOMA-IR and thyroid nodularity, even in pregnant women with the highest HOMA-IR values (≥4). This contrasts with the results reported by Yasar *et al.* (5) and Rezzonico *et al.* (40) for non-pregnant subjects. The absence of correlation between IR and thyroid nodules in our study may be attributed to methodological differences, population-specific characteristics or the impact of confounding factors. The group studied by Yasar *et al.* (5) was older (mean age of subjects with thyroid nodular disease (patients’ group) was 37.38 ± years, and of the control group, 34.83 ± years), and all subjects were iodine sufficient. It needs to be stressed that IR significantly impacted thyroid nodule size without affecting the number of lesions. In the study by Rezzonico *et al.* (40), IR was related to a higher frequency of thyroid nodular disease (both in obese and lean subjects), as well as with a larger number of nodules. However, subjects included in that study were also slightly older than our group (mean age 32 ± 7 years); the study group was relatively small and highly preselected (for example, women with abnormal thyroid gland palpation were excluded), the difference in ethnicity (South America) might also play a role. It needs to be noted that in both studies, no data on female subjects’ parity are available. It needs to be noted that not all studies confirmed the correlation between IR indices and thyroid nodularity. For example, Aydoğan *et al.* (42), in a small study comprising 50 patients with thyroid nodules and 50 healthy controls, also did not observe a correlation between HOMA-IR and the presence and number of thyroid nodules (again, in a non-pregnant group).

While interpreting our results, it needs to be remembered that pregnancy itself impacts TV and structure. Shokri *et al.* found that TV and nodularity in the first trimester of pregnancy were similar to those seen in women who were not pregnant (43). Fister *et al.* (44) linked TV increase with advancing pregnancy (BMI and TSH being its independent predictors). Pregnancy is linked to both the creation of new thyroid nodules and an increase in the size of existing thyroid nodules (45). Kung’s group reported an increased incidence of thyroid nodular disease at 3 months postpartum compared to the first trimester. 11.3% of investigated women developed new nodules as the pregnancy progressed. Pregnant women with thyroid nodules were also older (*P* < 0.01) and more obese (*P* < 0.02) than the women without thyroid nodules.

The other factor probably responsible for our negative findings on the relation between thyroid nodules and HOMA-IR in pregnant women is the young age of the study group. It is well known that thyroid nodular disease is more common as people age. As observed by Kwong *et al.* (46), the average number of nodules detected during initial assessment tends to rise from 1.5 in individuals aged 20–30 years to 2.2 in the oldest age groups (age >70 years).

## Limitations of the study

HOMA-IR is an indirect method for assessing IR with its inherent limitations. The gold standard for evaluating IR is the euglycemic hyperinsulinemic clamp. However, due to its complex nature, it is more commonly performed for research purposes than in clinical settings (47). To identify insulin-resistant individuals, it is crucial to have a clearly defined HOMA-IR cut-off value. So far, those have not been precisely determined, regardless of the studied populations (48, 49).

As mentioned before, it is important to note that IR or higher HOMA-IR values during pregnancy do not necessarily indicate impaired insulin sensitivity before conception. In the second half of pregnancy, reduced maternal insulin sensitivity should (at least to some extent) be considered a physiological phenomenon (which does not preclude its impact on thyroid function during gestation).

In our study, we operated on strictly defined data, with parameters such as TV, HOMA-IR and BMI measured only once at the time of recruitment. Therefore, our study groups differed in gestational age (the HOMA-nl subgroup comprised women in earlier pregnancy). We are aware that the results might be biased by the physiological impact of advancing pregnancy on the HOMA index and maternal BMI. We have tried to overcome that limitation by adjusting the results to pregnancy week.

In our study (as in most studies addressing thyroid function during gestation), we did not measure thyroxine-binding globulin (TBG), albumin and other protein concentrations during pregnancy, which may have impacted the accuracy of FT4 assays in serum samples. Human chorionic gonadotropin elevates estrogen levels during pregnancy, which raises TBG levels, resulting in increased binding of T4 to TBG and potentially causing a temporary decrease in freely circulating T4. Conversely, albumin levels decline during pregnancy due to a dilution effect resulting from an elevated total blood volume (50). Frequently employed FT4 tests, such as analog immunoassays, exhibit sensitivity towards variations in protein binding and may provide imprecise results (51). The precision of these tests depends on the balance between free and bound TH in the sample. However, the increased concentrations of TBG can disrupt this balance, leading to an underestimation of free T4 levels.

Although pregnancy is a risk factor for thyroid nodular disease, it is difficult to prove the role of IR (either physiological or pathological) in this phenomenon – particularly if thyroid structure and insulin sensitivity were not assessed before conception and longitudinally after delivery.

Regarding statistical analysis, possible limitations could arise from the distribution of observed data. As this is not a controlled study, data artifacts could negatively impact the accurate estimation of effects and relationships, particularly in the multiple regression models, where many quantitative variables had non-normal distributions and HOMA-IR was an imbalanced binary predictor. We addressed that through diagnostics and checks described in the statistical analysis section and feel confident about the validity of the analysis results.

## Conclusion

Insulin sensitivity should be considered a factor interplaying with thyroid function during pregnancy, with the reduced capability of adjusting to the increased TH demand with increasing IR indices. This phenomenon is generally independent of thyroid autoimmunity. Reduced insulin sensitivity may play a role in TV increase during pregnancy. However, its impact on thyroid nodularity seems negligible in the short term.

## Declaration of interest

The authors declare that there is no conflict of interest that could be perceived as prejudicing the impartiality of the work reported.

## Funding

The Polish National Programme for Elimination of Iodine Deficiency was funded by the Ministry of Health (coordinating institution: University Hospital in Krakow).

## Author contribution statement

AN conceived the concept of the study. AN, JP and MTM contributed to the design of the research. All authors were involved in data collection. AN, MTM and JP analyzed the data. All authors edited and approved the final version of the manuscript.
